# Oxalate nephropathy induced by octreotide treatment for acromegaly: a case report

**DOI:** 10.1186/1752-1947-6-215

**Published:** 2012-07-23

**Authors:** Karim Gariani, Sophie de Seigneux, Marie Courbebaisse, Marc Lévy, Solange Moll, Pierre-Yves Martin

**Affiliations:** 1 Department of Internal Medicine, Divison of General Internal Medicine, 4 rue Gabrielle-Perret-Gentil, 1211 Genève 4, Geneva, Switzerland; 2 Department of Medical Specialties, Nephrology Division, Geneva, Switzerland; 3 Service de Néphrologie et dialyse, Hôpital Tenon, APHP, Paris, France; 4 Department of Pathology, Clinical Pathology Division, Hôpitaux Universitaires de Genève, Geneva, Switzerland

**Keywords:** Oxalate nephropathy, Octreotide, Antibiotics, Oxalobacter formigenes

## Abstract

**Introduction:**

Oxalate nephropathy has various etiologies and remains a rare cause of renal failure. To the best of our knowledge, we report the first case of oxalate nephropathy following octreotide therapy.

**Case presentation:**

We report the case of a 78-year-old Caucasian man taking chronic octreotide treatment for acromegaly who presented with acute oxalate nephropathy after antibiotic therapy. The diagnosis was confirmed by urinary analysis and a kidney biopsy. The recovery of renal function was favorable after hydration and withdrawal of octreotide therapy.

**Conclusions:**

Oxalate nephropathy should be suspected in patients at risk who present with acute kidney injury after prolonged antibiotic treatment. This diagnosis should be distinguished from immuno-allergic interstitial nephritis and requires specific care. The evolution of this condition may be favorable if the pathology is identified correctly. Octreotide therapy should be considered a risk factor for enteric oxaluria.

## Introduction

Oxalate nephropathy is characterized by tubular deposition of calcium oxalate crystals due to either abnormal oxalate production or excretion. This condition can lead to acute and chronic tubular injury, interstitial fibrosis and progressive renal insufficiency. The etiologies of oxalate nephropathy are divided into primary and secondary hyperoxaluria. Primary hyperoxaluria is a rare autosomal recessive inherited metabolic disorder which leads to an increase in endogenous oxalate synthesis resulting in increased urinary oxalate excretion. Secondary hyperoxaluria is due to increased intestinal oxalate absorption, excessive dietary oxalate intake or excessive intake of oxalate precursors. Enteric hyperoxaluria due to malabsorption is principally caused by inflammatory bowel disease, jejuno-ileal bypass, short bowel syndrome, pancreatic insufficiency or alteration of oxalate degrading bacteria. More rarely, genetic alterations of intestinal oxalate transporters may be incriminated. Calcium plays a major role in the normal intestinal elimination of oxalate since it binds to oxalate in the intestinal lumen, thereby impeding its absorption. Malabsorption syndromes lead to increased oxalate absorption via two mechanisms: excessive non-absorbed fatty acid will chelate free calcium, resulting in increased free oxalate that will be reabsorbed and excreted by the kidney; in addition, fatty acids and dihydroxyl bile acids cause an increase in the permeability of the intestinal mucosa to oxalate also contributing to hyperoxaluria. This excess of oxalate urinary excretion may lead to either acute or chronic oxalate nephropathy.

We report a case of acute oxalate nephropathy associated with tubulointerstitial fibrosis in a patient treated for acromegaly with octreotide.

## Case presentation

A 78-year-old Caucasian man was admitted because of acute kidney injury to our hospital. His medical history was remarkable for acromegaly diagnosed 25 years ago that was first treated with radiotherapy. Due to a residual secretion of growth hormone he was then treated with octreotide for 10 years (10 mg every six weeks) with an adequate control of his insulin-like growth factor −1 (IGF-1) level. His medical history also revealed weight loss of 15 kg in the last four months without fever, dysuria, diarrhea or other symptoms.

Several months prior to admission, he was treated with sulfamethoxazole-trimethoprim and ciprofloxacin for a urinary tract infection. This treatment was followed by a partially regressive acute kidney injury that was attributed to immuno-allergic nephritis. No further investigations were undertaken at that time. His residual renal function was estimated to be 50 ml/minute/1.73 m^2^ according to the MRDR4 system.

During the weeks preceding admission a urinary culture yielded a multiresistant Enterobacteria which resulted in multiple successive antibiotic treatments (penicillin and two cephalosporins) over a period of five weeks. A rise in creatinine level was noted following these treatments. He was hospitalized for investigation of an acute kidney injury.

Upon hospital admission he was afebrile. His blood pressure was 120/70 mmHg and pulse rate was 72 per minute. His physical examination was unremarkable and there was no skin rash or lymphadenopathy

Laboratory studies were notable for a creatinine level of 4.9 mg/dL with conserved diuresis, resulting in stage F acute kidney injury according to the RIFLE (risk, injury, failure, loss, and end stage kidney disease (>three months))classification, a HCO_3_ level of 18.9 mM (22 to 29 mM), a phosphorus level of 6.59 mg/dL (normal 2.5 to 8.5 mg/dL) and a calcium level of 8.8 mg/dL (normal 8.5 to 10.5 mg/dL). He also had mild anemia. His leukocyte count was normal without eosinophilia. Urine dipstick showed traces of protein and hemoglobin. Immunologic tests were unremarkable and viral serologies were negative. Microscopic urine examination displayed four red cells M/L and 123 white cells with 3% of eosinophils. Urine cultures were positive for penicillin resistant citrobacter koseri. Abdominal ultrasound showed multiple cortical cysts of the left kidney, no pyelocaliceal dilatation and the presence of multiple gallstones.

**Figure 1:**
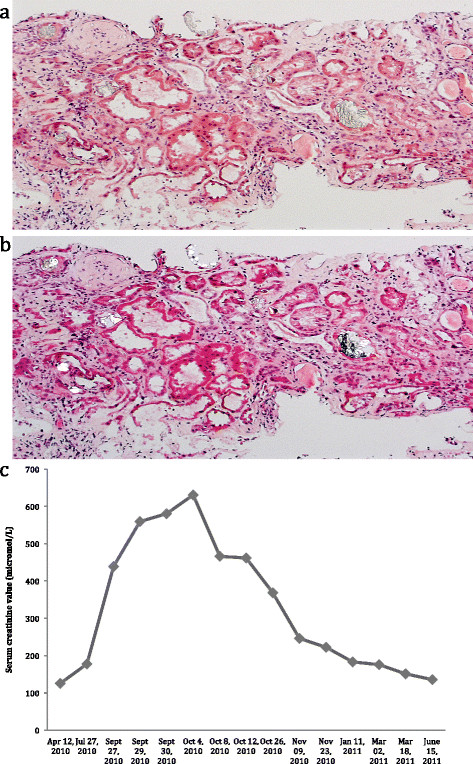
**a) diffuse tubular lesions with numerous oxalate crystals Hematoxylin and eosin (HE) staining; original magnification 130×. b)** diffuse tubular lesions with numerous oxalate crystals presenting birefringence under polarized light. **c)** Course of serum creatinine.

A renal biopsy was performed. Glomeruli displayed no abnormalities. Examination of the tubulointerstitial space revealed diffuse tubular atrophy with epithelial cell desquamation and necrosis as well as the presence of numerous intratubular and intracytoplasmic oxalate crystals presenting birefringrence under polarized light (Figure 1 a, b). Chronic interstitial nephritis characterized by an infiltration of lymphocytes and histiocytes was also seen. Immunofluoresence studies were negative. Infrared analysis of the biopsy confirmed the presence of whewellite, a rare form of crystallization of monohydrated calcium oxalate.

When asked again, the patient reported no history of vitamin C supplementation or excessive oxalate intake, no history of urinary tract stones and no relevant familial disease. During his hospital stay, no diarrhea was noted.

A complete urinary collection displayed elevated urinary oxalate excretion, with a low citrate level (Table 1). Primary hyperoxaluria was excluded as the urinary dosage of glycolate and l-glyceric acid were negative.

**Table 1:** Results of urinary collection performed upon hospital admission and ten months after cessation of octreotide therapy

**Parameters**	**During hospitalization**	**10 months after hospitalization and stopping octreotide therapy**	**Normal values**
**Fractional excretion of sodium (%)**	**10.1**	**1.07**	**<1**
**Fractional excretion of urea (%)**	**49.4**	**36.6**	**<35**
**UCa (micromol/24 hour)**	**2.24**	**1.2**	**2.5 to 7.5**
**UPh (micromol/24 hour)**	**5.4**	**19.8**	**13 to 42**
**UOx (micromole/24 hour)**	**1150**	**255**	**78 to 89**
**UGlycerate/Cr (mmol/mol)**	**7**		**<108**
**UGlycolate/Cr (micromol/mol)**	**2**		**<6**
**UCitrate/Cr (micromol/mol)**	**12**		**<457**
**UIsocitrate/Cr (micromol/mol)**	**8**		**<49**
**Plasma oxalate (micromole/L)**	**5**		**<33**

U, urinary.

A diagnosis of oxalate nephropathy with tubulointerstitial fibrosis secondary to enteric hyperoxaluria caused by octretotide and multiple antibiotic therapies was made.

Hydration was undertaken and octreotide therapy was stopped. Oral calcium and citrate supplementation were prescribed. Creatinine levels slowly decreased and he was discharged with a nephrologic follow up. The evolution of his condition was slowly favorable (Figure 1.c). A new urinary collection performed 10 months after withdrawal of octreotide therapy (Table 1) showed normal urinary oxalate excretion.

## Discussion

Hyperoxaluria leading to oxalate nephropathy is classified as primary or secondary and can manifest clinically by acute or progressive renal failure, nephrolithiasis and nephrocalcinosis. Primary hyperoxaluria is caused by a genetic defect in glycoxylate metabolism. Secondary hyperoxaluria results from varied enteric absorptive disorders such as pancreatic insufficiency, inflammatory bowel disease or jejuno-ileal bypass and also from high consumption of oxalate-rich food [1-4]

In the present case, octreotide that was administered for acromegaly probably had an important role in the pathogenesis of oxalate nephropathy. This synthetic somatostatin binds to somatotstatin-receptor subtypes 2, 3,and 5 and inhibits the release of 5-hydroxytryptamine (5-HT) and the secretion of insulin, glucagon, secretin, gastrin, vasoactive intestinal peptide (VIP), motilin, pancreatic polypeptide (PP), IGF-1 and growth hormone [5]. The most frequent side effects of octreotide are diarrhea, abdominal discomfort and gallstone formation in relation to inhibition of pancreatic exocrine secretions. In addition, inhibition of pancreatic secretions may cause steatorrhea and malabsoption leading to preferential binding of enteral calcium with fatty acids resulting in increased amounts of free oxalate for enteric absorption. Bile salts and malabsorbed fatty acids modify the colonic mucosa also leading to increased oxalate absorption [6]. These two phenomena result in an increased oxalate absorption followed by enhanced urinary oxalate excretion, and the formation of urinary calcium oxalate crystals.

A striking element in our patient’s history was his recent prolonged antibiotic treatment. Indeed the colonic flora contains *Oxalobacter formigenes*, a non pathogenic gram-negative anaerobic rod of the gastrointestinal tract. This bacterium plays a regulating role in oxalic acid absorption in the colon by using oxalate as its unique energy source. It not only degrades oxalate but also creates a transepithelial gradient favorable to colonic secretion of oxalate. The destruction of this organism increases the enteral absorption of oxalate leading to an increased excretion of urinary oxalate. A direct association between antibiotic consumption and the absence of *O. formigenes* in the gastrointestinal tract has been reported. Several cases of hyperoxaluria on long-term antibiotic treatments have been recorded, mostly in patients already at risk of hyperoxaluria, such as patients suffering from pancreatic insufficiency or inflammatory bowel disease. [7-9].

Our patient suffered from two episodes of acute kidney injury shortly after the use of different antibiotics. It is likely that an alteration in the bacterial flora caused by the antibiotic therapy dramatically enhances oxalate enteric absorption leading to acute oxalate nephropathy in a patient with chronic malabsorption related to chronic octreotide treatment, as indicated by the gall stones and weight loss. The evolution of this condition was favorable following hydration and withdrawal of antibiotic and octreotide treatments, resulting in normalization of oxalate urinary excretion.

## Conclusions

This case highlights the fact that acute kidney injury in the context of antibiotics should evoke the differential diagnosis of oxalate nephropathy in susceptible patients and that octreotide should be considered a risk factor for enteric hyperoxaluria.

## Consent

Written informed consent was obtained from the patient for publication of this manuscript and any accompanying images. A copy of the written consent is available for review by the Editor-in-Chief of this journal.

## Competing interests

The authors declare that they have no competing interests.

## Authors’ contributions

KG compiled and analyzed the patient’s data and wrote the initial draft. SDS and PYM corrected the draft. SDS and ML were treating physicians for the patient. SM and MC performed the histological examination of the kidney. All authors read and approved the final manuscript.

## References

[B1] Hoppe B Beck BB Millinier DS The primary hyperoxalurias Kidney Int 2009 75 1264 1271 10.1038/ki.2009.32 19225556 PMC4577278

[B2] Cartery C Faquer S Karras A Cointault O Buscail L Modesto A Ribes D Rostaing L Chauveau D Giraud P Oxalate nephropathy associated with chronic pancreatitis Clin J Am Soc Nephrol 2011 6 1895 1902 10.2215/CJN.00010111 21737848 PMC3359534

[B3] Nasr SH D’Agati VD Said SM Stokes MB Largoza MV Radhakrishnan J Markowitz GS Oxalate nephropathy complicating Roux-en-Y gastric bypass: an underrecognized cause of irreversible renal failure Clin J Am Soc Nephrol 2008 2 1676 1683 10.2215/CJN.02940608 PMC2572276 18701613

[B4] Nasr SH Kashtanova Y Levchuk V Markowitz GS Secondary oxalosis due to excess vitamin C intake Kidney Int 2006 70 1672 10.1038/sj.ki.5001724 17080154

[B5] McKeage K Cheer S Wagstaff AJ Octreotide long-acting release (LAR): a review of its use in the management of acromegaly Drugs 2003 63 2473 2499 10.2165/00003495-200363220-00014 14609359

[B6] Stauffer JQ Hyperoxaluria and intestinal disease. The role of steatorrhea and dietary calcium in regulating intestinal oxalate absorption Am J Dig Dis 1977 22 921 928 10.1007/BF01076170 920694

[B7] Sidhu H Hoppe B Hesse A Tenbrock K Brömme S Rietschel E Peck AB Absence of Oxalobacter formigenes in cystic fibrosis patients: a risk factor for hyperoxaluria Lancet 1998 352 1026 1029 10.1016/S0140-6736(98)03038-4 9759746

[B8] Mittal RD Kumar R Bid HK Mittal B Effect of antibiotics on Oxalobacter formigenes colonization of human gastrointestinal tract J Endourol 2005 19 102 106 10.1089/end.2005.19.102 15735393

[B9] Lefaucheur C Hill GS Amrein C Haymann JP Jacquot C Glotz D Nochy D Acute oxalate nephropathy: a new etiology for acute renal failure following nonrenal solid organ transplantation Am J Transplant 2006 6 2516 2521 10.1111/j.1600-6143.2006.01485.x 16889602

